# Enhanced hypocrellin production via coexpression of alpha-amylase and hemoglobin genes in *Shiraia bambusicola*

**DOI:** 10.1186/s13568-018-0597-0

**Published:** 2018-05-02

**Authors:** Ruijie Gao, Huaxiang Deng, Zhengbing Guan, Xiangru Liao, Yujie Cai

**Affiliations:** Key Laboratory of Industrial Biotechnology, School of Biotechnology, Jiangnan University, 1800 Lihu Road, Wuxi, 214122 Jiangsu China

**Keywords:** Hypocrellin, Coexpression, Amylase, Hemoglobin, Solid state fermentation, *Shiraia* sp. SUPER-H168

## Abstract

*Shiraia bambusicola* is an important and valuable macrofungus and hypocrellins are its main secondary metabolites which have been widely applied in many medical fields. However, during SSF process of this filamentous fungus, use ratio of corn substrate and dissolved oxygen supply are two main limiting factors, which influence production cost, yield and product quality. To solve these problems, overexpressions of *amy365*-*1* and *vgb* in *S. bambusicola* were investigated and three overexpression transformants were constructed. Results demonstrated that expressions and coexpression of AMY365-1 and VHb not only increased the productions of biomass, amylase, hypocrellin, but also up-regulated relative expression levels of four central carbon metabolism genes (*pdc*, *ald*, *acs*, *acc*) and seven hypocrellin biosynthesis genes (*fad*, *mono*, *zftf*, *omef*, *msf*, *pks*, *mco*). Furthermore, expression of VHb decreased SSF period. When *amy365*-*1* and *vgb* were coexpressed, relative expression levels of *zftf* and *pks* reached their highest levels at 72 h under liquid fermentation, hypocrellin production reached the highest level 75.85 mg/gds which was 2.99-fold compared with wild type strain within 11 days under SSF, and residual starch of solid substrates was decreased from 35.47 to 14.57%.

**Electronic supplementary material:**

The online version of this article (10.1186/s13568-018-0597-0) contains supplementary material, which is available to authorized users.

## Introduction

*Shiraia bambusicola* is an important and valuable macrofungus in the medical and food industries. Hypocrellins are the main secondary metabolites of *S. bambusicola*, including hypocrellin A, hypocrellin B, hypocrellin C and hypocrellin D (Fang et al. 2006), a type of perylenequinone. Under illumination, hypocrellins react with oxygen and generate reactive oxygen species (ROS), including singlet oxygen (^1^O_2_) and the superoxide radical. Abundant ROS cause cellular oxidative stress and kill cells by damaging cellular macromolecules, including lipids, DNA and proteins (Trachootham et al. 2008). Based on this characteristic, hypocrellins have been widely applied in many medical fields, such as photodynamic tumor therapy and antivirus treatments (Gao et al. 2012; Jin et al. 2013). They also have been used to treat skin diseases, gastric diseases and some vascular diseases. In addition to benefiting the pharmaceutical industry, hypocrellins also have extensive potential applications in the agricultural, cosmetic, food, and feed industries (Shen et al. 2012; Su et al. 2010, 2011). So far, yield of hypocrellin from natural extraction was too low to meet it current medical demand. Therefore, improvement of hypocrellin production was needed to meet its demand.

In our previous work, we isolated a high-yield hypocrellin producing strain, *Shiraia* sp. SUPER-H168 (Liang et al. 2009); hypocrellins produced by *Shiraia* sp. SUPER-H168 under solid state fermentation (SSF) have been isolated and identified. At the same time, corn was found to be the best substrate after evaluating eight kinds of agro-industrial crops and residues (Cai et al. 2010). Compared with liquid state fermentation, SSF offers numerous advantages which include high volumetric productivity and relatively higher concentration of the products. SSF was found to be the best fermentation of industrialized production for hypocrellin with *S. bambusicola*. Cracked corn was produced from maize endosperm of which starch is the major constituent. Normal corn starch consists of about 75% branched amylopectin and about 25% amylose, that is linear or slightly branched (Sandhu and Singh 2007). In previous studies, residual starch of dry fermented substrate remained at a high level, and hypocrellin production was increased when external amylases were added (data was not showed in this study). In order to improve the yield of hypocrellin and increase the use ratio of corn substrate, an α-amylase gene *amy365*-*1* (GenBank accession no. *MF535183*) from *Shiraia* sp. SUPER-H168 was overexpressed.

Furthermore, during SSF process of the filamentous fungus, the dissolved oxygen supply is a limiting factor, which influences growth, yield, protein production, product quality and production cost (Mora-Lugo et al. 2015; te Biesebeke et al. 2006). *Vitreoscilla* hemoglobin (VHb) is a soluble homodimeric protein, which is synthesized by aerobic Gram-negative bacterium *Vitreoscilla* (Wakabayashi et al. 1986) and functions as an oxygen carrier and transporter (Bonaventura et al. 2013). VHb expression in heterologous hosts can improve oxidative metabolism, cell growth, protein synthesis, bioremediation and metabolite production (Heerd et al. 2012; Hofmann et al. 2009; Li et al. 2016a, b; Mora-Lugo et al. 2015; Stark et al. 2011, 2015; Zhang et al. 2014; Zhu et al. 2011). To the best of our knowledge, VHb has not been used in *S. bambusicola* to date. Therefore, the *vgb* gene engineering in *Shiraia* sp. SUPER-H168 was explored and the effects of VHb on growth and hypocrellin production were investigated.

In this study, overexpressions of *amy365*-*1* and *vgb in S. bambusicola* were investigated, respectively. Furthermore, coexpression of *amy365*-*1* and *vgb* was also investigated. Related genes of hypocrellin biosynthesis and enzymes in central carbon metabolism for efficient conversion of sugars into acetyl-CoA were investigated. Related genes of hypocrellin biosynthesis included FAD/FMN-containing dehydrogenase gene (*fad*), salicylate 1-monooxygenase gene (*mono*), zinc finger transcription factor gene (*zftf*), *O*-methyltransferase gene (*omef*), major facilitator superfamily gene (*msf*), polyketide synthase gene (*pks*), multicopper oxidase gene (*mco*) which were reported by Deng et al. (2016a). Hypocrellin which is a polyketide is synthesized from one molecule of acetyl-CoA and six molecules of malonyl-CoA (Deng et al. 2016a; Newman and Townsend 2016). Four genes of enzymes in central carbon metabolism which convert pyruvate to acetyl-CoA and malonyl-CoA were also investigated. They are pyruvate decarboxylase gene (*pdc*), acetaldehyde dehydrogenase gene (*ald*) and acetyl-CoA synthetase gene (*acs*) and acetyl-CoA carboxylase gene (*acc*).

## Materials and methods

### Materials

Corn was purchased from local supermarket (self-brand, Wuxi, Jiangsu, China).

### Microorganism and medium

*Shiraia* sp. SUPER-H168 (CCTCC M 207104) was a stock culture of the Laboratory of Biochemistry, School of Biotechnology, Jiangnan University, Wuxi, Jiangsu Province, China (Liang et al. 2009). *Shiraia* sp. SUPER-H168 was routinely maintained on PDA slants at 4 °C by regular sub-cultivation (no longer than 3 months). *Escherichia coli* strain DH5α (Takara, Dalian, China) was used as a host for cloning, and was grown in Luria–Bertani medium at 37 °C.

Regeneration medium: 20 g/L glucose, 1 g/L K_2_HPO_4_, 0.5 g/L MgSO_4_∙7H_2_O, 0.5 g/L KCl, 0.01 FeSO_4_∙7H_2_O, 3 g/L yeast extract, 10 g/L peptone, 200 g/L potato extract, and 0.06% TritonX-100.

Seed culture medium (50 mL) which was potato dextrose medium (Liang et al. 2009) was carried out in a Erlenmeyer flask (250 mL).

Liquid fermented medium was as follows: 5% corn flour, 1% yeast extract, 228 mg/L K_2_HPO_4_, 174 mg/L K_2_SO_4_, 294 mg/L CaCl_2_, 492 mg/L MgSO_4_∙7H_2_O, 0.1 mg/L ZnSO_4_, 0.08 mg/L CuSO_4_ and 0.06% Tween 80.

Solid-state medium was carried out in a Erlenmeyer flask (250 mL) with 40 g cracked corn, and then moistened with salt solution which included glucose (1.65%, w/w, g/g dry substrate) and NaNO_3_ (0.43%, w/w) until 50% initial moisture contents.

### Inoculum preparation

A spore suspension was obtained as follows: *Shiraia* sp. SUPER-H168 was grown on PDA slants in dark at 30 °C for 7 days. The black massive spores were harvested from the surface by pouring sterile distilled water and scraping the spores and homogenized aseptically in a Sorvall Ominimixer for 10 min. The spore concentration was measured by counting with a hemacytometer under microscope (Shi et al. 2009). The spore suspension was used to inoculate the subsequent fermentation immediately. Seed culture was inoculated with 3.5 mL spore suspension (10^6^ spores mL^−1^) (Cai et al. 2010) and cultured on a rotary shaker at 200 rpm and 30 °C for 48 h.

### Liquid state fermentation

Liquid culture (50 mL) in a Erlenmeyer flask (250 mL) was inoculated with 3.5 mL seed culture and cultured on a rotary shaker at 200 rpm and 30 °C for 96 h. Wild type *Shiraia* sp. SUPER-H168 was chosen as the control strain for analysis of biomass, hypocrellin production, residual sugar and relative expression levels. Hypocrellin was produced, extracted and detected as described by Cai et al. (2011). RNA was isolated from different strains at 48, 72 and 96 h.

### Solid state fermentation

Fermentation was done at 30 °C for 15 days under relative humidity higher than 95%. The optimized SSF conditions were as follows: substrate particle size 0.8–1 mm, initial moisture content 50%, and temperature 30 °C. The optimum compositions of the supplementary glucose and NaNO_3_ were 1.65 and 0.43% (w/w), respectively (Cai et al. 2010). The samples were removed at regular intervals of 48 h after agitation and were analyzed for different parameters. Fermented substrate was dried at 60 °C and pulverized to pass through a 0.2-mm sieve. One gram sample was refluxed with 50 mL methanol in a water bath at 70 °C for 2 h. The extract was filtered through 0.45-μm membrane filter and diluted to 100 mL. After that, hypocrellins was determined by reverse-phase high-performance liquid chromatography (HPLC) (Cai et al. 2010).

### RNA isolation and first strand cDNA synthesis

RNA was isolated using a Takara RNA Extraction Kit (Takara, Ōtsu, Japan) according to manufacturer’s protocol. To eliminate genomic DNA, RNA samples were treated with RNase-free DNase I (Takara). The quantity and quality of the total RNA extracted was determined using a NanoDrop-2000C spectrophotometer (Thermo Scientific, Wilmington, DE, USA) and the integrity was evaluated by analyzing the ratio between rRNA subunits of 18 S and 28 S after electrophoresis. The first strand cDNA was synthesized by reverse transcribing 500 ng RNA with 5× All-In-One RT MasterMix (Applied Biology Materials Inc., Richmond, Canada), and cDNA samples were stored at − 20 °C (Deng et al. 2016b).

### Codon optimization and gene synthesis

Amino acid sequence of VHb (GenBank accession no. *AAA75506*) and corresponding nucleotide sequence of *vgb* gene were shown in Additional file 1: Figure S1. After codon optimization, the optimized *vgb* gene (GenBank accession no. *MG735184*) was synthesized by GENEWIZ, Inc in Soochow (Jiangsu, China). Alignment between original (GenBank accession no. *KM108313.1*) and optimized *vgb* gene sequences was shown in Additional file 1: Figure S1. Alpha-amylase gene *amy365*-*1* was cloned when cDNA sample of *Shiraia* sp. SUPER-H168 was used as template.

### Plasmid construction

**Fig. 1:**
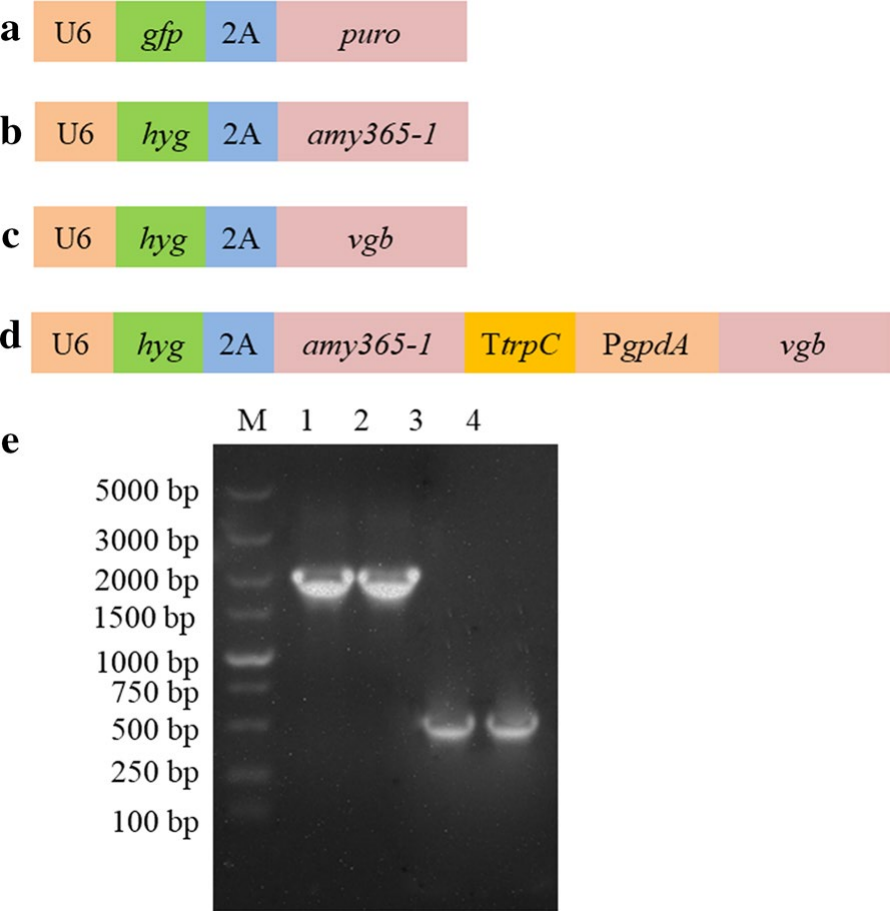
Construction of expression vectors and cDNA of *amy365*-*1*, *vgb* in this study. **a** Pgfppuro, **b** Phygamy365-1, **c** Phygvgb and **d** PhygPgpdAamy365-1-vgb. U6, U6 promoter; PgpdA, gpdA promoter; Ttrpc, the *trpC* terminator; *hyg*, gene of protein resistant to hygromycin B; 2A, nucleotide sequence encoding 2A peptide which acted as a coexpression linker; *amy365*-*1*, α-amylase gene; *vgb*: gene of *Vitreoscilla* hemoglobin. **e** cDNA of *amy365*-*1* and *vgb*. Lane M: DL5000 DNA Marker; lanes 1–2: cDNA of *amy365*-*1*; lanes 3–4: cDNA of *vgb*

A lentiviral expression vector, Pgfppuro, obtained from Applied Biological Materials (ABM) Inc. (Richmond, Canada), was chosen as an expression plasmid in *S. bambusicola* which had been confirmed by Deng et al. (2016a). The vector consists of a U6 promoter, green fluorescent protein gene *gfp*, nucleotide sequence encoding 2A peptide, and gene of the protein resistant to puromycin *puro* (Fig. 1a). On the basis of Pgfppuro, Phyg expression vector and PhygPgpdA coexpression vector were constructed in this study. The 2A oligopeptide is emerging as a highly effective new tool for the facile coexpression of multiple proteins in a single transformation step, whereby a gene encoding multiple proteins, linked by 2A sequences, is transcribed from a single promoter (de Felipe et al. 2006). 2A functions has already been applied to coexpress GFP and Hyg selective markers in *S. bambusicola* as described by Deng et al. (2016a).

The Phyg expression vector was constructed by the following process. First, the gene *hyg* of Hyg protein was used to replace the *gfp* of the Pgfppuro expression plasmid. The Pgfppuro vector was linearized through amplification reaction with VF1 and VR1 primers. The Hyg protein gene *hyg* was amplified from pUCATPH plasmid with primers hygF and hygR. The amplified *hyg* with 15-bp nucleotides (the underlined sequences in Table 1) was reverse-complemented with the linearized lentiviral vector. On the basis of the 15-bp reverse-complement nucleotides between linearized lentiviral vector and amplified *hyg*, the ligation-free cloning kit (ABM Inc., Richmond, Canada) was used to construct Phyg vector by homologous recombination.

**Table 1:** Primers used for construction of overexpression plasmid

Primer symbol	Primers (5′-3′)
VF1	GGTGGCACTAGAGAAGACTATTTTTG
VR1	GAATTCGGAAGCGGGCAGTG
hygF	TTCTCTAGTGCCACCATGAAAAAGCCTGAACTCACCG
hygR	CCCGCTTCCGAATTCTTCCTTTGCCCTCGGAC
VF2	GGATCCGGGGCCGGGGTTGCTC
VR2	ACGCGTTCCGGAAATCAACCTCTGG
amy365-1F	CCCGGCCCCGGATCCATGTCGGCTCCTCGTCCACGAGGC
amy365-1R	ATTTCCGGAACGCGTTTATTGCCATGTACTATTCACACTCGAAGCC
vgbF	CCCGGCCCCGGATCCATGCTTGATCAACAAACGATCAACA
vgbR	ATTTCCGGAACGCGTCTATTCGACGGCTTGGGCGT
TtrpCF	ATCCACTTAACGTTACTGAAATCATC
TtrpCR	CATTTTTCGACAATCCGAGTGGAGATGTGGGAG
PgpdAF	GATTGTCGAAAAATGTAAAGG
PgpdAR	GATGAAGTGTTTGTTGAGCTAGGGGAG
amy365-1R1	TAACGTTAAGTGGATTTATTGCCATGTACTATTCACACTCGAAGCC
vgbF1	AACAAACACTTCATCATGCTTGATCAACAAACGATCAACA

In order to overexpress *amy365*-*1*, overexpression plasmid Phygamy365-1 (Fig. 1b) was constructed. The gene of PURO protein was replaced by *amy365*-*1*. It was constructed in the same way as construction of Phyg vector with linearized plasmid which was amplified with primers VF2 and VR2 and *amy365*-*1* which was amplified with primers amy365-1F and amy365-1R. Phygvgb (Fig. 1c) was also constructed in the same way as Phygamy365-1. *vgb* was amplified with primers vgbF and vgbR.

In order to coexpress *amy365*-*1* and *vgb,* coexpression plasmid PhygPgpdAamy365-1-vgb (Fig. 1d) was constructed. It was consisted of the following five fragments (in order): (1) linearized vector amplified from plasmid Phyg with primers VF2 and VR2; (2) *amy365*-*1* was amplified with primers amy365-1F and amy365-1R1; (3) the *TtrpC* terminator amplified from pAN52-1Not (GeneBank Accession No. Z32524) with primers TtrpCF and TtrpCR; (4) the *PgpdA* promoter was amplified from genomic DNA of *S. bambusicola* with primers PgpdAF and PgpdAR. *PgpdA* promoter had been identified and chosen as the native promoter for overexpression in *S. bambusicola* (Deng et al. 2017). (5) *vgb* was amplified with primers vgbF1 and vgbR. They were integrated by homologous recombination, and then PhygPgpdAamy365-1-vgb was constructed. All primers used in this study and reverse-complement nucleotides with underlined were showed in Table 1.

### Preparation of protoplasts and fungal transformation

The procedures for fungal protoplast generation and transformation were carried out as described by Deng et al. (2016a). Spore was selected as the original host and the cell wall was eliminated after 4-h degeneration with enzyme mixture. Plasmid (5 μg) was added to 200 μL of protoplasts in STC solution (1.2 M sorbitol, 10 mM Tris–HCl and 10 mM CaCl_2_ at pH 7.5), and 100 μL of cold PTC buffer (50% PEG 3350, 10 mM Tris–HCl and 10 mM CaCl_2_ at pH 7.5) was then added to the mixture. The mixture was incubated on ice for 20 min. Then, 800 μL of PTC added to the mixture and kept at room temperature for 15 min, followed by centrifugation at 5000*g* and 4 °C for 5 min. The supernatant was then discarded. Protoplasts were suspended in regeneration medium and incubated with shaking at 75 rpm and 30 °C until the cell walls were recovered. Then protoplasts were spread onto the regeneration agar medium with 500 μg/mL of hygromycin B.

### Real-time quantitative PCR (RT-qPCR)

The relative expression levels of hypocrellin biosynthesis genes which included *fad*, *mono*, *zftf*, *omef*, *msf*, *pks*, *mco*, and related genes in central carbon metabolism which included *pdc*, *ald*, *acs*, *acc* were studied. To standardize the relative quantification of the cDNA, *Shiraia* sp. SUPER-H168 18S rRNA gene was selected as an endogenous control gene. Primers used for RT-qPCR analysis of hypocrellin biosynthesis genes and related genes in central carbon metabolism were showed in Table 2.

**Table 2:** Primers and relevant information of reference and target genes

Gene symbol	Gene name	Primers (5′-3′)
*amy365*-*1*	α-amylase	F: TGGATTACGCTACTTATTAC
		R: GTATTGCTAACGGTATTCA
*vgb*	*Vireoscilla* hemoglobin	F: ATCGTCGGTCAAGAACTT
		R: CCCAGGCATCAAGGATAT
*fad*	FAD/FMN-containing dehydrogenase	F: TGTGACCGCCATCACCTTAC
		R: TTGTCGTATGGGTGGGAAGC
*Mono*	Salicylate 1-monooxygenase	F: TCTCGGGGAATTATGGCACG
		R: ACAACCGTTCTCGCATCAGT
*zftf*	Zinc finger transcription factor	F: GAACACCGTCGCAAGATTCG
		R: TCATTGGCATCGCTTGGAGT
*omef*	*O*-methyltransferase	F: GAACTACCTGAAGGCACGCT
		R: GCTCGGAAGGATACTCGCTC
*msf*	Major facilitator superfamily	F: TCCCGTAGCCTTGCTTTCTG
		R: CCGGCTTCTTCTTGACGCTA
*pks*	Polyketide synthase	F: TGCTGAGGTAGCAGTCAAGC
		R: TTATGCTACGGTCGTCGCTC
*mco*	Multicopper oxidase	F: TATGGCGCTACGAGTGGAC
		R: ACTCCCTGGCCGATAACGTA
*pdc*	Pyruvate decarboxylase	F: ATTGTAACGAACTGAATGCT
		R: GTGGTGACTATGGCTGAA
*ald*	Acetaldehyde dehydrogenase	F: GTTGGCAGTGAGAATGGA
		R: CTGTTGCGTAGTTGATGATG
*acs*	Acetyl-CoA synthetase	F: GTTGGCTTATACGCTCAA
		R: TTCTGGAATCATAGGTAGGT
*acc*	Acetyl-CoA carboxylase	F: ATCTCAACTGCCGAATACA
		R: AGTGCCAACAATCTCCAA
18S rRNA	18S rRNA gene	F: GAAAGTTAGGGGATCGAAGA
		R: TAGTCGGCATAGTTTACGGT

The amplification efficiency (E) of all primer sets was tested with serial dilutions of template cDNA and calculated from the slope of the dilution curve according to the equation:

$$ {\text{E}} = \left[ { 10^{{ - ( 1/{\text{slope}})}} - 1} \right] \times 100\% . $$

The expressions of related genes were assessed in hyphae grown in liquid fermented media. RT-qPCR reactions were carried out in 96-well block with a CFX96 Real-Time PCR Detection System (Bio-Rad, Hercules, CA, USA). The thermal profiles were performed using the following conditions: 95 °C for 10 min, 30 cycles of 95 °C for 3 s, 57 °C for 30 s, 72 °C for 30 s. In order to evaluate the specificity of primer sets used for RT-qPCR amplification, the melting curve was analyzed. Every amplification was performed in triplicate.

Relative expression levels of genes were calculated based on the threshold cycle (C_T_) deviation of the treated sample versus a control and expressed in comparison to reference gene. In this study, wild type strain was selected as a control. Since wild type had no vgb, C_T_ of vgb in wild type was set as 30

$$ {\text{Relative}}\,{\text{expression}}\,{\text{ratio}} = 2^{{\left[ {\Delta {\text{CT}}\left( {\text{control}} \right) - \Delta CT\left( {\text{treated}} \right)} \right]}} . $$

### Residual sugar analysis

Residual sugar analysis was performed by the amyloglucosidase/α-amylase method (AOAC method 996.11) with the total starch assay kit from Megazyme (Wicklow, Ireland). Under SSF, cracked corn and solid substrates were dried at 80 °C for 24 h before analysis (Bluhm and Woloshuk 2005). The residual sugar was also detected under liquid state fermentation.

## Results

In order to increase the use ratio of corn substrate and improve the dissolved oxygen supply, α-amylase gene *amy365*-*1* and *Vitreoscilla* hemoglobin gene *vgb* were overexpressed, and three plasmids which included Phygamy365-1, Phygvgb and PhygPgpdAamy365-1-vgb were constructed and three transformants were obtained.

### Liquid state fermentation

Three overexpression vectors were constructed and their transformants were cultured using corn flour as carbon source. Wild type *Shiraia* sp. SUPER-H168 was chosen as the control strain for analysis of biomass, hypocrellin production, residual sugar and relative expression levels.

**Fig. 2:**
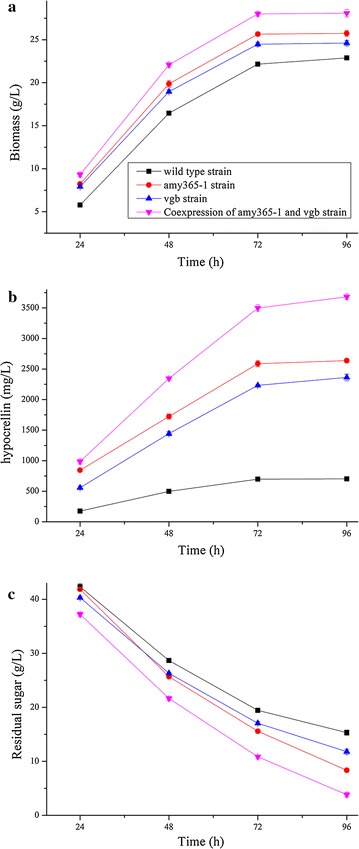
Biomass (**a**), hypocrellin production (**b**) and residual sugar (**c**) cultured under liquid state fermentation by three overexpression transformants and wild type strain. The error bars represent standard deviation of triplicate measurements. Standard deviations were under 14 per cent of the averages

Biomass (Fig. 2a) and hypocrellin production (Fig. 2b) were both gradually increased in four strains, at the same time, residual sugar was gradually decreased (Fig. 2c). Three overexpression transformants all had positive effects on biomass and hypocrellin production at different culture times. In addition, hypocrellin production in co-expression strain of AMY365-1 and VHb reached the highest level 3681 mg/L at 96 h, it was 5.24-fold compared with wild type strain (703 mg/L at 96 h). Hypocrellin productions of two overexpression strains *amy365*-*1* strain and *vgb* strain reached 2638 and 2362 mg/L at 96 h, respectively. Biomass and residual sugar in co-expression strain of AMY365-1 and VHb reached 28.08 and 3.83 g/L at 96 h, respectively. While biomass and residual sugar in wild type strain reached 22.89 and 8.35 g/L at 96 h, respectively.

### RNA validation

RNA samples isolated from four strains were assessed for integrity and quality. All absorbance ratios at 260/280 nm of RNA samples ranged from 1.8 to 2.0. Agarose gel electrophoresis revealed no degradation.

### RT-qPCR analysis of hypocrellin biosynthesis genes and related genes in central carbon metabolism

**Fig. 3:**
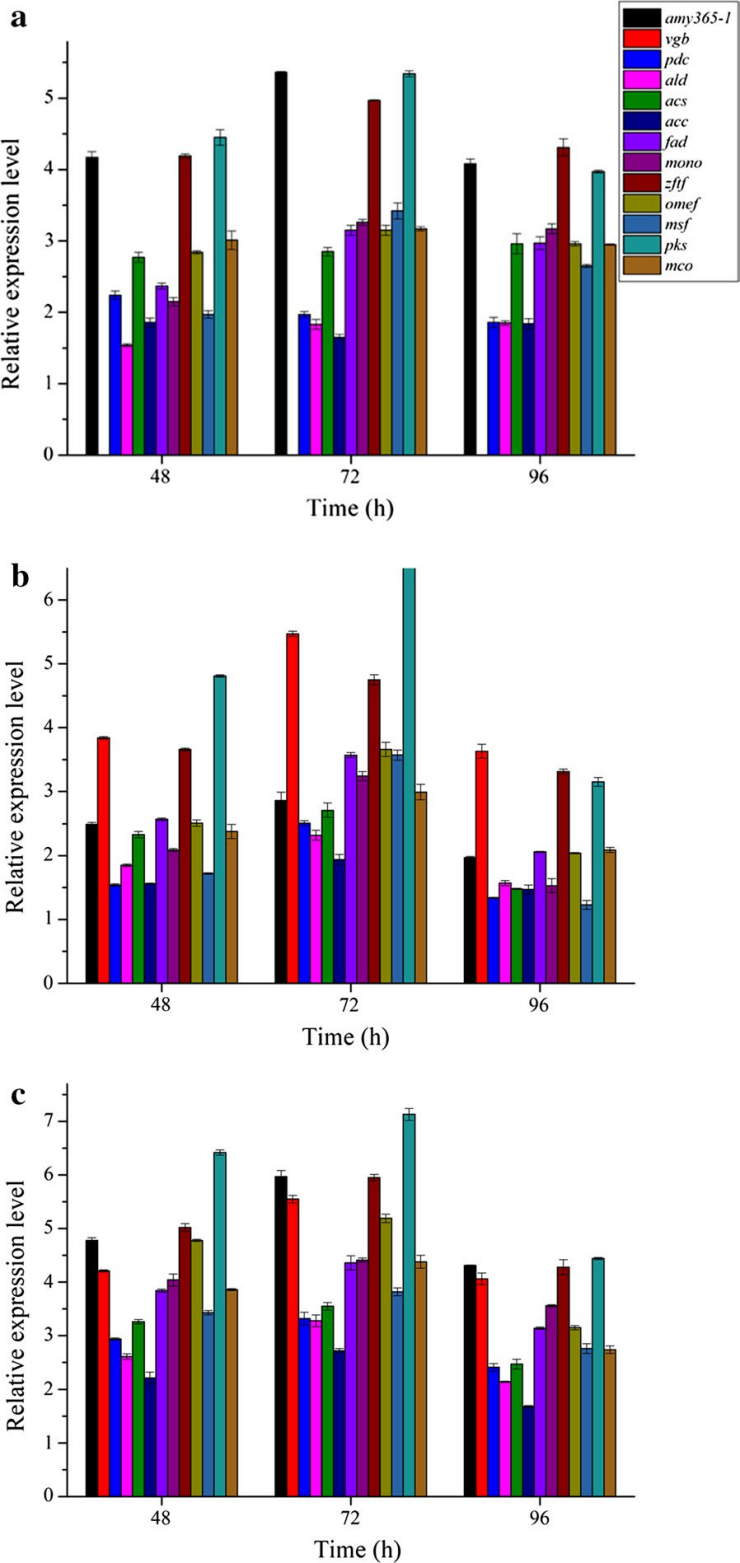
Relative expression levels of *amy365*-*1*, *vgb*, seven hypocrellin biosynthesis genes and four related genes in central carbon metabolism when cultured on corn flour. **a** Phygamy365-1, **b** Phygvgb and **c** PhygPgpdAamy365-1-vgb. The error bars represent standard deviation of triplicate measurements. Standard deviations were under 7 per cent of the averages

In order to analyze the effects of AMY365-1 and VHb overexpression on genes of hypocrellin biosynthesis, relative expression levels of α-amylase gene *amy365*-*1*, hemoglobin gene *vgb*, seven hypocrellin biosynthesis genes including *fad*, *mono*, *zftf*, *omef*, *msf*, *pks*, *mco* and four related genes in central carbon metabolism including *pdc*, *ald*, *acs*, *acc* in three overexpression transformants were studied at 48, 72 and 96 h (Fig. 3). Data of *amy365*-*1* strain (Fig. 3a), *vgb* strain (Fig. 3b) and coexpression of *amy365*-*1* and *vgb* strain (Fig. 3c) were shown, respectively.

The relative expression levels of all the studied genes were increased in three overexpression strains compared with the wild type strain, which meant that there was a positive correlation among *amy365*-*1*, *vgb*, hypocrellin biosynthesis genes and related genes in central carbon metabolism. Relative expression levels of seven hypocrellin biosynthesis genes were all increased when AMY365-1 and VHb were overexpressed, especially those of *zftf* and *pks* were increased clearly in three overexpressed strains. Relative expression levels of *zftf* and *pks* reached their highest levels in PhygPgpdAamy365-1-vgb constructed strain at 72 h, they are 5.98 fold and 7.07-fold compared with wild type strain, respectively. Relative expression level of *amy365*-*1* was increased when VHb was overexpressed (Fig. 3b, c). Relative expression level of *acs* also was increased clearly compared with other *pdh* and *acc* which related to central carbon metabolism in three overexpressed strains. In addition, hypocrellin biosynthesis genes reached their highest relative expression levels in PhygPgpdAamy365-1-vgb constructed strain at 72 h. The results agreed with hypocrellin production in liquid state fermentation.

### Solid state fermentation

**Fig. 4:**
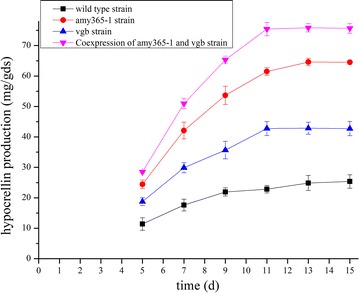
Hypocrellin production cultured under SSF by three overexpression transformants and wild type strain. The error bars represent standard deviation of triplicate measurements. Standard deviations were under 10 per cent of the averages

Hypocrellin productions under SSF were also studied in four strains (Fig. 4). Under SSF, hypocrellin productions were all increased in three overexpression strains compared with wild type strain. In PhygPgpdAamy365-1-vgb constructed strain, hypocrellin production reached 75.85 mg/gds within 11 days which was the highest yield in all studied, and it was 2.99-fold compared with wild type strain (25.37 mg/gds). Hypocrellin productions in Phygamy365-1 constructed strain and Phygvgb constructed strain reached 64.59 mg/gds with 13 days and 42.75 mg/gds with 11 days, respectively. When VHb was overexpressed, SSF period was reduced to 11 days in Phygvgb constructed strain and PhygPgpdAamy365-1-vgb constructed strain. At 15 d, residual starch of fermented substrates which was cultured by PhygPgpdAamy365-1-vgb constructed strain was 14.57%, compared with that of wild type strain was 35.47%.

## Discussion

*Shiraia bambusicola* has demonstrated recently its potential for hypocrellin production in fermentation systems and particularly in SSF (Liang et al. 2009). Furthermore, corn was found to be the best substrate after evaluating eight kinds of agro-industrial crops and residues (Cai et al. 2010). Starch is the main constituent in corn, and it also is the main carbon source when hypocrellin was produced under SSF with *S. bambusicola*. In addition, it is generally assumed that there is a limitation in the oxygen supply to the cells that are in close contact with the substrate in solid-state cultures with filamentous fungi (Oostra et al. 2001; Rahardjo et al. 2005). The use ratio of corn substrate and oxygen supply were two key aspects which influenced the yield of hypocrellin under SSF. In order to produce hypocrellin at an industrial scale in the future, specific α-amylase AMY365-1 and VHb were selected and overexpressed in *Shiraia* sp. SUPER-H168. Three transformants included Phygamy365-1 strain, Phygvgb strain and PhygPgpdAamy365-1-vgb strain were constructed.

This study showed that *S. bambusicola* harboring *amy365*-*1* yielded higher amounts of biomass and hypocrellin in comparison to wild type strain. The overexpression and function of AMY365-1 result in more efficient hydrolysis of corn starch. Presumably, hypocrellin production was largely dependent on starch catabolism, which accelerate carbon metabolism and ensure the timely and effective supply of glucose in cell. Glycolysis is a central pathway for the catabolism of carbohydrates which converts glucose to two pyruvate molecules. The pathway from pyruvate to acetyl-CoA and malonyl-CoA may be enhanced because relative expression levels of *pdc*, *ald*, *acs* and *acc* were increased when *amy365*-*1* was overexpressed. With respect to hypocrellin biosynthesis genes, relative expression levels of *zftf* and *pks* were both increased when *amy365*-*1* was overexpressed. ZFTF is a global transcription factor that mediated gene cluster of secondary metabolite (Yin and Keller 2011), hypocrellin production was increased along with the relative expression level of *zftf* increased. PKS (Deng et al. 2016a) was a key enzyme that catalyzed hypocrellin biosynthesized via repetitive decarboxylative claisen condensation of acetyl-CoA and malonyl-CoA. The relative expression level of *pks* was also increased along with that of *zftf*, and this is the main reason why hypocrellin increased.

Furthermore, this study also showed that *S. bambusicola* harboring *vgb* yielded higher amounts of biomass, amylase, and hypocrellin in comparison to those of wild type strain. The effects of *amy365*-*1* overexpression and *vgb* expression and the combination of the two on expression of *amy365*-*1* gene are not simple additive. Maybe it was due to the facts that AMY365-1 was an inducible amylase and relative expression level of *amy365*-*1* was increased when VHb was overexpressed. The results are in good agreement with previous reports of fungi, where increased levels of biomass and metabolite yields were associated with heterologous expression of VHb. For instance, threefold elevated levels of α-amylase and a 31% increase in the total secreted protein by VHb-expressing *Schwanniomyces occidentalis* under oxygen-limiting environment (Suthar and Chattoo 2006), increased biomass, glucose uptake rate, glycerol production rate, specific oxygen uptake rate and CO_2_ production rate by *Aspergillus niger* (Hofmann et al. 2009), improved biomass and protein production by *Aspergillus sojae* (Mora-Lugo et al. 2015), increased biomass and arachidonic acid production by *Mortierella alpine* (Zhang et al. 2017), these results were all obtained when engineering these filamentous fungi with VHb. The presence of VHb did not significantly affect cell growth in *Ganoderma lucidum* (Li et al. 2016b) and *Pseudomonas aeruginosa* (Geckil et al. 2001). The results suggest that the function of VHb may vary in different organisms and under different growth conditions. The biosynthesis of hypocrellin is an energy-requiring process. Energy efficiency is the major constraint for the improvement of hypocrellin production, because ATP is needed during central carbon metabolism and conversion from acetyl-CoA to malonyl-CoA. The present and function of VHb in *S. bambusicola* may result in more efficient energy production, which in turn may have positively effects on central carbon metabolism and hypocrellin biosynthesis.

Relative expression levels of genes which involved central carbon metabolism (*pdc*, *ald*, *acs*, *acc*) and hypocrellin biosynthesis (*fad*, *mono*, *zftf*, *omef*, *msf*, *pks*, *mco*) were all increased in three transformants compared with wild type strain. The results in *vgb* strain are in good agreement with previous reports which showed that presence of VHb may regulate the expression of host genes (Stark et al. 2015). For instance, the transcription levels of genes *pgm*, *ugp*, and *gls* in the polysaccharide biosynthesis were enhanced in *vgb*-bearing *Ganoderma lucidum* (Li et al. 2016b). The transcriptional levels of the key genes in the electron transfer chain, TCA cycle and welan gum synthesis pathway were enhanced in *vgb*-bearing *Sphingomonas* sp. HT-1 (Liu et al. 2017). In this study, expressions of AMY365-1 and VHb both increase the activity of transcription factor ZFTF which is a global transcription factor that mediated gene cluster of secondary metabolite.

In other fungi, *Cercospora nicotianae* which produced cercosporin and *Fusarium verticillioides* which produced fumonisin B_1_ were both have a zinc finger transcription factor in their polyketide biosynthetic gene cluster. Hypocrellin and cercosporin are all polyketide with polyketomethylene backbone. The core gene cluster for cercosporin biosynthesis includes genes encoding a polyketide synthase, two *O*-methyltransferases, monooxygenase, an MFS transporter, three oxidoreductases and a Zn(II)_2_Cys_6_ transcription regulator (CTB8). CTB8 transcriptional activator controls cercosporin production by controlling gene transcript levels. (Chen et al. 2007). Additionally, *FUM21*, which encodes a Zn(II)_2_Cys_6_ transcription factor, is present in the FUM gene cluster, and is required for the transcriptional activation of FUM genes and fumonisin biosynthesis (Brown et al. 2007; Visentin et al. 2012). Among fungi, members of the Cys-2 His-2 (C2H2) TF family are predicted to regulate pathogenicity, cell differentiation, carbon utilization, and development (Park et al. 2008). Carbon availability is a key environmental factor regulating FB_1_ biosynthesis in *F. verticillioides*. Regardless, signal transduction pathways regulating FB_1_ biosynthesis in response to carbon metabolites are not completely understood (Kim et al. 2011). In this study, expressions of AMY365-1 and VHb both increase the activity of transcription factor ZFTF. Signal transduction pathways regulating ZFTF in response to carbon and oxygen are not completely understood, and detailed investigations must be performed at molecular level in the future.

Although many encouraging results have been obtained in bacteria, yeast and filamentous fungi, mechanism of VHb action and regulation should be further elucidated. The presence and function of VHb in enhancing respiration and formation of ATP has been considered to be the reason for improvements of cell growth, protein synthesis and metabolite production (Stark et al. 2011). Therefore, the *vgb* gene engineering in *Shiraia* sp. SUPER-H168 serves as a very effective tool for enhancing growth, respiration, protein synthesis, and metabolism at low exogenous oxygen concentrations especially penetrative hyphae under SSF by promoting oxygen delivery. The mechanism of VHb on aerobic metabolism has not been fully known. To explain the VHb mechanism in *S. bambusicola*, detailed investigations must be performed at molecular level in the future.

In conclusion, α-amylase AMY365-1 and VHb were both successfully overexpressed and coexpressed in *Shiraia* sp. SUPER-H168. They both resulted in an improvement of biomass, amylase synthesis, hypocrellin production and up-regulation of relative expression levels of central carbon metabolism genes and hypocrellin biosynthetic genes. At the same time, expression of VHb resulted in a reduction of SSF period. This study reflects the potentials of α-amylase and VHb to enhance hypocrellin production under SSF at an industrial scale in the future.

## Additional file

**Additional file 1: Figure S1.** Codon optimization of vgb gene. The first line, amino acid sequence of VHb; the second line, original nucleotide sequence of *vgb* gene; the third line, optimized sequence of *vgb* gene, changed nucleotides are shown as highlighted with yellow color.

## Abbreviations

ROS: reactive oxygen species
SSF: solid state fermentation
VHb: *Vitreoscilla* hemoglobin
*fad*: FAD/FMN-containing dehydrogenase gene
*mono*: salicylate 1-monooxygenase gene
*zftf*: zinc finger transcription factor gene
*omef*: *O*-methyltransferase gene
*msf*: major facilitator superfamily gene
*pks*: polyketide synthase gene
*mco*: multicopper oxidase gene
*pdc*: pyruvate decarboxylase gene
*ald*: acetaldehyde dehydrogenase gene
*acs*: acetyl-CoA synthetase gene
*acc*: acetyl-CoA carboxylase gene
PDA: potato dextrose agar
RT-qPCR: real-time quantitative PCR
