# Supplementary material for: Enhanced hypocrellin production via coexpression of alpha-amylase and hemoglobin genes in Shiraia bambusicola
Source: AMB Express. 2018 May 2;8:71. doi: 10.1186/s13568-018-0597-0 (PMC5931956; doi:10.1186/s13568-018-0597-0)
Supplement: Supplementary file 1 — Additional file 1: Figure S1. Codon optimization of vgb gene. The first line, amino acid sequence of VHb; the second line, original nucleotide sequence of vgb gene; the third line, optimized sequence of vgb gene, changed nucleotides are shown as highlighted with yellow color. [file 13568_2018_597_MOESM1_ESM.docx]

**Appendix A. Additional data for:**

**Enhanced hypocrellin production via coexpression of alpha-amylase and hemoglobin genes in *Shiraia bambusicola***

Ruijie Gao, Huaxiang Deng, Zhengbing Guan, Xiangru Liao, Yujie Cai*

Key Laboratory of Industrial Biotechnology, School of Biotechnology, Jiangnan University, 1800 Lihu Road, Wuxi, Jiangsu 214122, China

Ruijie Gao

E-mail address: [gaoruijie1989@163.com](mailto:gaoruijie1989@163.com)

Huaxiang Deng

E-mail address: [289682921@qq.com](mailto:289682921@qq.com)

Zhengbing Guan

E-mail address: [guanzb@jiangnan.edu.cn](mailto:guanzb@jiangnan.edu.cn)

Xiangru Liao

E-mail address: [xrliao@jiangnan.edu.cn](mailto:xrliao@jiangnan.edu.cn)

* Corresponding author: Yujie Cai

Telephone number: +86-18961727911

E-mail address: [yjcai@jiangnan.edu.cn](mailto:yjcai@jiangnan.edu.cn)


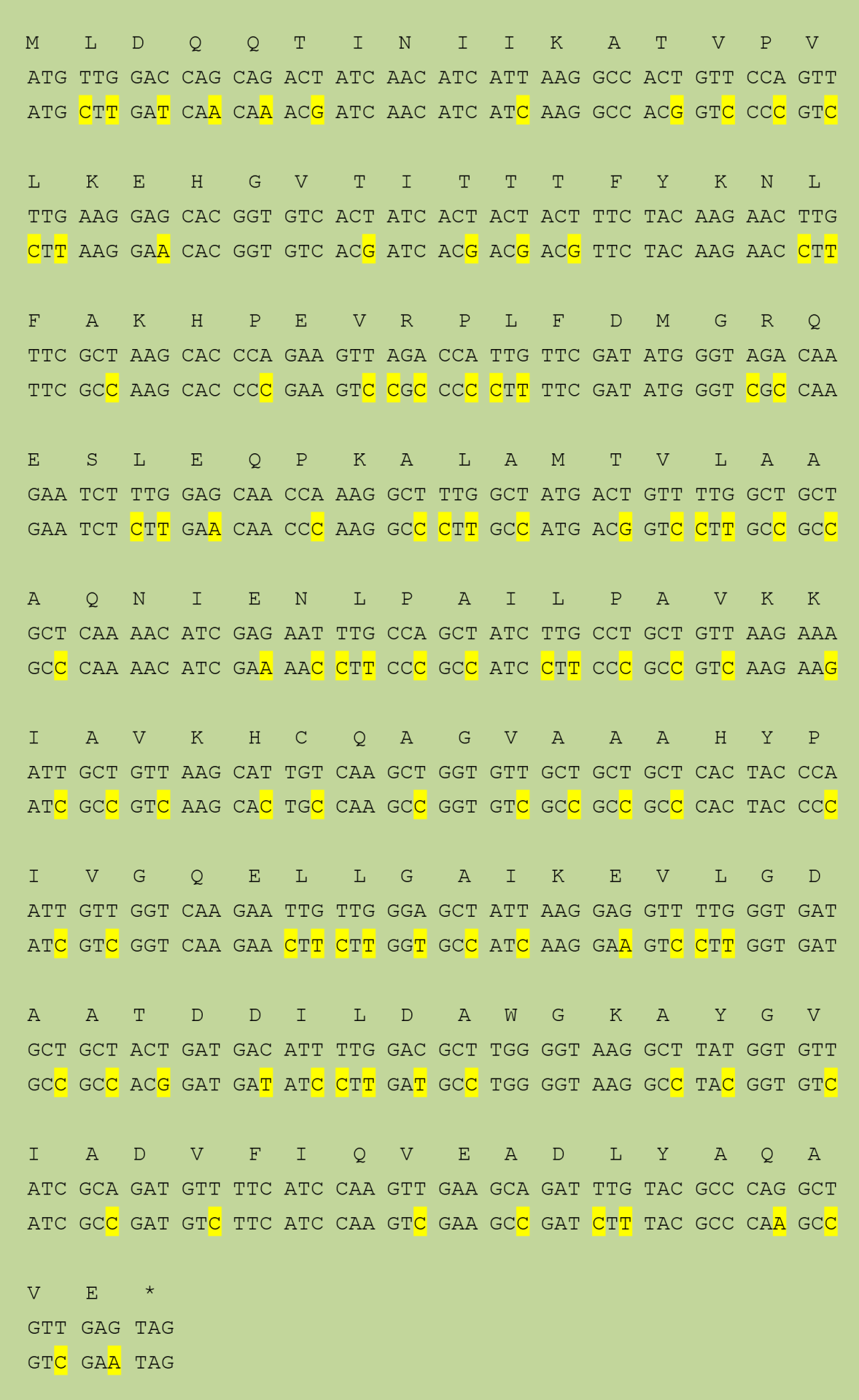


**Fig. S1 Codon optimization of vgb gene.** The first line, amino acid sequence of VHb; the second line, original nucleotide sequence of *vgb* gene; the third line, optimized sequence of *vgb* gene, changed nucleotides are shown as highlighted with yellow color.
